# Dosimetric evaluation of rectum and bladder using image-based CT planning and orthogonal radiographs with ICRU 38 recommendations in intracavitary brachytherapy

**DOI:** 10.4103/0971-6203.39417

**Published:** 2008

**Authors:** Swamidas V. Jamema, Sherly Saju, Umesh Mahantshetty, S. Pallad, D. D. Deshpande, S. K. Shrivastava, K. A. Dinshaw

**Affiliations:** Department of Medical Physics, Tata Memorial Hospital, Mumbai, India; 1 Department of Radiation Oncology, Tata Memorial Hospital, Mumbai, India

**Keywords:** Carcinoma of uterine cervix, ICRU 38 rectal and bladder point doses, image-based dosimetry, intracavitary brachytherapy

## Abstract

The purpose is to compare CT-based dosimetry with International Commission on Radiation Units and Measurements (ICRU 38) bladder and rectum reference points in patients of carcinoma of uterine cervix treated with intracavitary brachytherapy (ICA). Twenty-two consecutive patients were evaluated. Orthogonal radiographs and CT images were acquired and transferred to PLATO planning system. Bladder and rectal reference points were identified according to ICRU 38 recommendations. Dosimetry was carried out based on Manchester system. Patient treatment was done using ^192^Iridium high dose rate (HDR) remote after-loading machine based on the conventional radiograph-based dosimetry. ICRU rectal and bladder point doses from the radiograph plans were compared with D_2_, dose received by 2 cm^3^ of the organ receiving maximum dose from CT plan. V_2_, volume of organ receiving dose more than the ICRU reference point, was evaluated. The mean (±standard deviation) volume of rectum and bladder was 60 (±28) cm^3^ and 138 (±41) cm^3^ respectively. The mean reference volume in radiograph and CT plan was 105 (±7) cm^3^ and 107 (±7) cm^3^ respectively. It was found that 6 (±4) cm^3^ of rectum and 16 (±10) cm^3^ of bladder received dose more than the prescription dose. V_2_ of rectum and bladder was 7 (±1.7) cm^3^ and 20.8 (±6) cm^3^ respectively. Mean D_2_ of rectum and bladder was found to be 1.11 (±0.2) and 1.56 (±0.6) times the mean ICRU reference points respectively. This dosimteric study suggests that comparison of orthogonal X-ray-based and CT-based HDR ICA planning is feasible. ICRU rectal point dose correlates well with maximum rectal dose, while ICRU bladder point underestimates the maximum bladder dose.

Carcinoma of the uterine cervix is the most common cancer among women, where a combination of external beam radiotherapy (EBRT) and intracavitary brachytherapy (ICA) constitutes the main stay of treatment, especially in the advanced stages. Brachytherapy forms an integral part of radiation therapy and forms the cornerstone for both the control rates and toxicities.[1,2] ICA offers the advantage of delivering a very high dose to the tumor, with low doses to adjacent critical structures, namely, bladder and rectum. Quantification of doses received by bladder and rectum is very important as they are the dose-limiting structures in ICA. The dose delivered to the critical structures from ICA are difficult to quantify accurately.

Conventionally, dosimetry of ICA is carried out using orthogonal radiographs where point doses to critical structures are calculated according to the ICRU 38[3] recommendations. But the point doses may not represent the dose received by the volume of the organs. It has been reported that the ICRU point doses do underestimate doses received by the rectum and bladder.[4–7] The ratio of maximum dose to the rectum and bladder from the CT planning to that obtained from radiograph-based planning has a wide range.[4–6] Over the previous decade, there have been significant advances in imaging and three-dimensional image-based brachytherapy planning. The advantage of three-dimensional image-based brachytherapy planning is the accurate quantification of doses received by various volumes of surrounding critical structures. Traditional methods of treatment planning have yielded high control rates of the tumor and acceptable complications of the normal tissues. However, a more accurate understanding of the doses received by the dose-limiting structures may contribute in improving the therapeutic ratio, both in terms of treatment outcome and reducing the complications further. In addition, with three-dimensional planning, it may be possible to evaluate the dose-volume response relationship by assessing the composite doses of both EBRT and ICA.

In order to document, validate and compare volume-based doses to rectum and bladder with the conventional standard ICRU 38 rectal and bladder points, we undertook this dosimetric study.

## Materials and Methods

Patients with FIGO stage IIB or IIIB of carcinoma of uterine cervix treated between May 2004 and February 2005 with radical radiation therapy and high dose rate (HDR) ICA were studied. Twenty-two applications consisting of 12 IIB and 10 IIIB of HDR ICA were analyzed. Four patients of IIB and 6 patients of IIIB were included in this study. All the patients were treated with standard dose of external beam radiotherapy, followed by ICA-HDR brachytherapy according to the institutional protocol. All the HDR ICA applications were carried out under general anesthesia. Each application consisted of placement of an intrauterine tandem (4-6 cm) into the uterine cavity after minimal dilatation and the ovoids (diameter of ovoids: 1.5-2.5 cm) in the vagina at the level of fornices. This was followed by radiopaque gauze packing in both anterior and posterior vaginal space to displace the bladder and rectum away from the vaginal applicators and to fix the applicator positions. A dose of 7 Gy was prescribed at point A. According to the institutional protocol, patients with stage IIB receive five applications once weekly, starting from second week onwards; whereas patients with stage IIIB receive three applications starting from 3^rd^-4^th^ week of external radiation.

### Intracavitary brachytherapy dosimetry

#### A. Conventional dosimetry:

Orthogonal radiographs (anterior-posterior and lateral) were taken on a conventional simulator (Varian, Palo Alto, USA) with radiopaque markers in the applicators. Orthogonal radiographs were reconstructed and the treatment planning was done using PLATO planning system (Brachytherapy v14.3, Nucletron, Veneendal, The Netherlands). Source positions were loaded according to the standard loading pattern in accordance with the Manchester system. Point A was defined on the radiographs as being 2 cm superior to the flange and 2 cm lateral from the axis of the intrauterine tandem. Bladder and rectal reference points were identified according to ICRU 38[3] recommendations. In addition to the ICRU rectal reference point, two additional rectal points were defined at 1 cm superior and inferior to the ICRU rectal reference point based on our earlier report.[8] Dwell positions were optimized to minimize the dose to rectal and bladder points. The dose was prescribed to point A, treatment was carried out using ^192^Iridium HDR remote after-loading machine (Nucletron, Veneendal, The Netherlands) based on the conventional radiograph-based dosimetry.

#### B. CT-based ICA-HDR dosimetry:

All the above intracavitary applications were simultaneously taken up for CT planning. CT scans of 5-mm slice-thickness were obtained using a Siemens Somatom Emotion scanner, 4 cm above the tandem superiorly and to the level of anus inferiorly. Rectum and bladder were delineated. Rectum was contoured from recto-sigmoid junction superiorly till ischial tuberosity inferiorly. The entire bladder was contoured. Treatment planning was carried out using PLATO (Brachytherapy v 14.3, Nucletron, The Netherlands) planning system. Point A, ICRU rectal and bladder reference points were identified on CT planning. For each application, the corresponding optimized source positions used in radiograph-based planning were duplicated for CT image-based planning. Reconstruction of metal applicators using CT images was difficult due to the presence of artifacts. For quality assurance reasons, the accuracy of reconstruction was evaluated by overlaying the isodose distribution of the radiograph-based planning with the CT planning. Shift in point A, location of dwell positions in tandem and ovoids with respect to the applicator origin (flange) were evaluated. A variation of ±2 mm shift was set as acceptability criteria.

### Evaluation: Conventional vs. CT-based dosimetry correlation

Cumulative dose volume histogram (cDVH) was calculated for every plan with 25 mm margins around the implanted volume in all directions with 100,000 calculation points randomly placed in the volume of interest. Reference volume recommended by ICRU 38[3] was evaluated in both radiograph-based planning and CT planning. Total reference air kerma (TRAK) was used to calculate reference volume in both radiograph-based plan and in CT plan.[9] The comparison and correlation of doses to bladder and rectum was carried out using D_2_, V_2_ and the ratio of D_2_ to the ICRU reference point doses.

## Results

All the 22 applications, done with both conventional and CT-based planning, were eligible for comparison. The activity of the ^192^iridium source used for the patient treatment varied between 3.0 and 9.1 Ci. The total reference air kerma was an average of 0.41 ± 0.04 cGy at 1 m.

### Dose to point A

The mean reference volume in radiograph plan and CT plan was found to be 105 ± 7 cm^3^ and 107 ± 7 cm^3^ respectively. Dose to point A from CT planning and radiograph-based planning were compared. The results showed that the correlation between point A dose of these two plans was found <5% in 14 patients, 5-10% in 6 patients and >10% in 2 patients. The largest deviation was found to be 11.7%. The mean volumes receiving doses 50% (3.5 Gy), 100% (7.0 Gy), 150% (10.5 Gy) and 200% (14 Gy) for both 2D radiograph-based plan and CT plan were evaluated [Table 1].

**Table 1:** Volumes of various isodoses from orthogonal radiograph-based plan and image-based computerized tomography plan

*Dose in Gy (% of prescription)*	*Volume (standard deviation) from orthogonal plan in cm^3^*	*Volume (standard deviation) from CT plan in cm^3^*
3.5 (50)	305 (±8)	309 (±9)
7.0 (100)	105 (±7)	107 (±7)
10.5 (150)	56 (±3)	57 (±3)
14 (200)	33.9 (±2)	35 (±2)

### Correlation of rectum and bladder reference points

**Figure 1:**
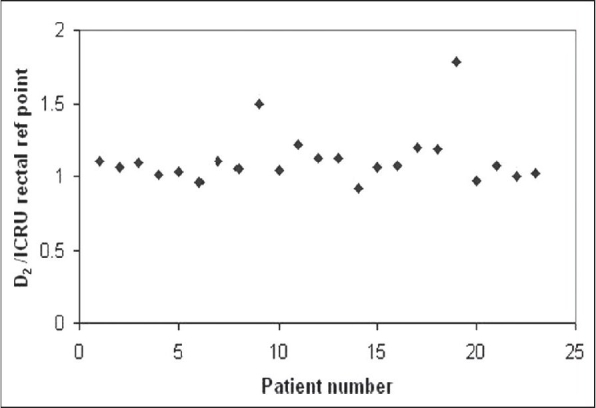
Ratio of D_2_, the dose received by the 2 cm^3^ of the volume of rectum, calculated from image-based CT planning to ICRU rectal reference point calculated from the orthogonal radiograph-based plan for all patients

**Figure 2:**
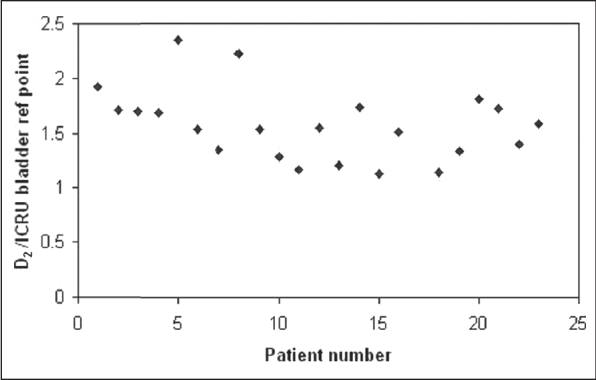
Ratio of D_2_, the dose received by the 2 cm^3^ of the volume of bladder, calculated from image-based CT planning to ICRU bladder reference point calculated from the orthogonal radiograph-based plan for all patients

The mean (±standard deviation) contoured volume of rectum and bladder was 60 (±28) cm^3^ and 138 (±41) cm^3^ respectively. The mean D_2_ of rectum and the ICRU rectal points was 5.16 Gy and 4.63 Gy, while for bladder it was 7.12 Gy and 4.56 Gy respectively. Mean D_2_ of rectum and bladder was found to be 1.11 (±0.2) and 1.56 (±0.6) times the mean ICRU reference points respectively. V_2_ for rectum and bladder was found to be 7 (±1.7) cm^3^ and 20.8 (±6) cm^3^ respectively. It was found that 6 (±4) cm^3^ of rectum and 16 (± 10) cm^3^ of bladder received dose more than the prescription dose to point A. Figures 1 and 2 show the ratios D_2_ to the ICRU rectal and bladder points for each application. Table 2 summarizes the results of the mean doses of ICRU rectal and bladder points obtained from the radiograph-based planning with the maximum doses received from the CT plans.

**Table 2:** Dose received by international commission on radiation units reference point from orthogonal radiograph-based plan and D2 from image-based CT plan for bladder and rectum

*Organ*	*Volume cm^3^*	*D_2_ Gy*	*Dose to ICRU point Gy*	*V_2_ cm^3^*	*D_2_/Dose to ICRU ratio*
Rectum	60 (±28)	5.16 (±1.22)	4.63 (±1.27)	7 (±1.7)	1.11 ± (0.2)
Bladder	138 (±41)	7.12 (±1.87)	4.56 (±1.44)	20.8 (± 6)	1.56 ± (0.6)

D_2_ is the dose received by the 2 cm^3^ of the volume of the critical structure receiving maximum dose in CT-based planning, and V_2_ is the volume of the critical structure receiving dose more than the ICRU reference point dose. Values given in parenthesis are for standard deviation

## Discussion

**Figure 3:**
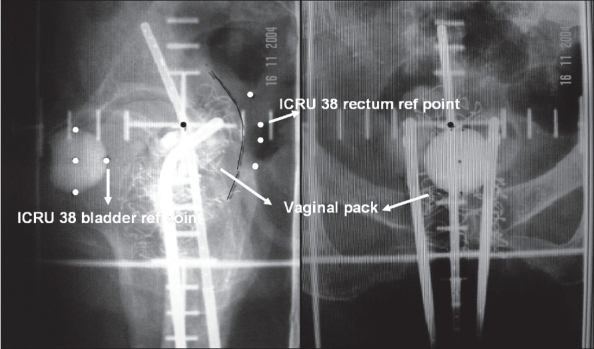
ICRU rectal and bladder reference points are marked on orthogonal radiographs. Rectal reference points are taken 5 mm posterior to the vaginal wall, which could be visualized from the radiopaque gauze packing

Traditionally, dosimetry of ICA for carcinoma cervix was based on orthogonal radiographs [Figure 3] with ICRU 38recommendations, which allow the evaluation of point doses such as Manchester points A, B, ICRU rectal and bladder reference points. Orthogonal radiographs provide spatial information of the applicator with respect to bony structures. However, this time-tested system has a limitation of computing the doses received by the volumes of the critical structures. Over the past two decades, there have been significant advances in imaging and volume-based brachytherapy planning, with an advantage to determine the dose volume parameters for the critical structures. We undertook this dosimetric study to compare, validate and document the correlation between volume - based doses to rectum and bladder with the conventional standard ICRU 38 rectal and bladder points.

ICRU 38 recommends the reporting of reference volume which can be obtained from the product of height, width and the thickness of the pear-shaped isodose volume. Esche *et al.*[10] evaluated the reference volume from the milligram-hours radium. Other investigators[5,11–13] calculated using the product of height, width and thickness of the pear-shaped volume. However, in the present study, reference volume was calculated from TRAK, which was based on our previous report.[9] As expected, the reference volumes in both radiograph-based plan and the CT plans were almost similar in our study.

**Figure 4:**
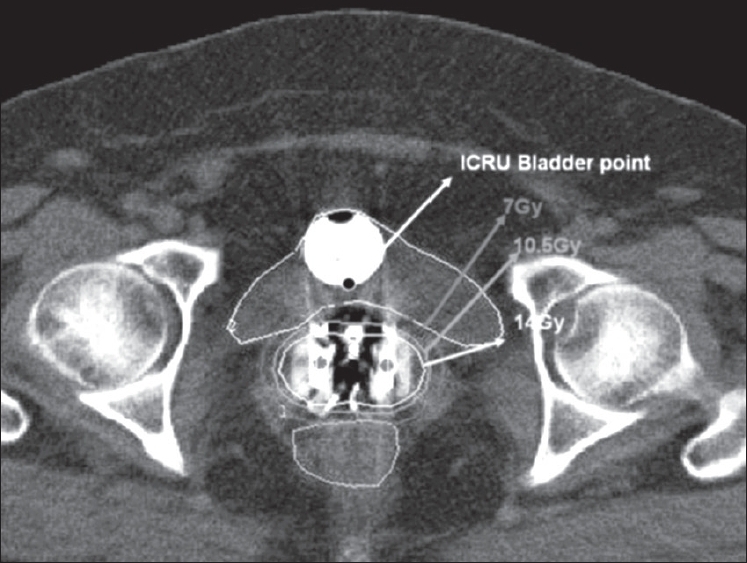
Dose distribution of image-based CT planning for a representative patient

The dose to ICRU rectal point from the radiograph-based planning was almost similar to that of CT-based planning [Figure 4]. Mean D_2_ of rectum was found to be 1.11 ± 0.2 times the mean ICRU rectal reference point, suggesting that there was no significant difference between the radiograph-based ICRU rectal point and CT-based estimation of the parameter D_2_. Studies[5,14–16] had shown poor correlation of rectal doses from the CT plans with the ICRU rectal reference point dose. ICRU rectal reference point underestimated the maximum dose, and the ratio of the maximum dose and the ICRU rectal dose reported varies in the range of 1.4-2.8.[5,14–16] The large variations reported may be attributed to several factors such as different types of applicators used, inter-observer variability in contouring of critical structures, etc. Saarnak *et al.*[17] reported significant inter-observer variability in the contouring of critical structures in the patients treated with ICA for carcinoma cervix. Further, van den Bergh *et al.*[18] suggested that a good correlation between ICRU rectal point from radiograph-based planning and the maximum dose from the CT planning could be obtained by calculating the maximal dose to the rectal wall as it could be better visualized on the axial section of the CT images. Pelloski *et al.*[6] reported almost similar results as that of the present study for rectum (Pelloski: 1.00; present study: 1.11 ± 0.2). In our study, the use of radiopaque gauze pack in the vagina enabled accurate definition of ICRU rectal point and contouring of anterior rectal wall and hence there was good correlation.

For bladder, the results of the present study suggest that the ICRU bladder reference point does not correlate with the maximum dose from the CT planning [Figure 4]. Mean D_2_ of bladder was found to be 1.56 ± 0.6 times the mean ICRU bladder reference point. These results agree with the other studies published in the literature, where the ICRU bladder reference point underestimated the maximum dose by two to three times.[12,15,16,19] Barillot *et al.*[12] found that the maximum dose in the bladder calculated from the trans-abdominal ultrasonography was an average 2.7 times higher than the ICRU bladder reference point. Good correlation was found between ICRU bladder reference doses calculated by ultrasonography and orthogonal radiographs. However, no correlation was found between the ICRU reference dose and the maximal bladder dose. Out of 69 cases evaluated, in 75% of patients the maximum dose exceeded the ICRU reference dose by 2-8 times.[12] The following authors also evaluated the ratio of maximum dose to the ICRU bladder reference point dose: Kapp *et al.,*[14] 1.44 (range 1.0-1.7); Tan *et al.,*[20] 1.32 (range 0.62-2.43); Fellner *et al.,*[5] 1.4 ± 0.5; and the present study, 1.56 ± 0.3. ICRU 38 bladder point underestimates the bladder doses, and this finding has been consistent with those of many series mentioned above. However, the wide range of the ratio could be attributed to the various methods used, such as ultrasonography, radiographs and CT, to evaluate the maximum dose. However, the ratios found using the CT images[5,6,14,20] were found to be in good agreement with each other and that found in the present study.

In image-based dosimetry, reconstruction of applicators was done using the CT images. Ling *et al.*[15] minimized the metal artifacts by manipulating the CT window and level settings for standard Fletcher Suit applicators during the CT scan. Fellner *et al.*[5] had followed the method of overlaying the isodose distribution calculated on the basis of radiographs on the CT images with the help of corresponding points (coordinate transformation method). Pelloski *et al.*[6] reconstructed the metal Fletcher Suit applicators on the CT images, and the accuracy of reconstruction and source localization was evaluated by comparing the distances of certain points with the expected values (from the orthogonal radiographs). In the present study, reconstruction of the applicators using the CT images was difficult due to the artifacts produced by the metal applicators. Hence to evaluate the accuracy of reconstruction for quality assurance reasons, the CT reconstruction was compared with the orthogonal radiograph-based reconstruction of the applicator. The visualization of applicators in the orthogonal radiographs was of excellent quality, and it formed the baseline for comparison. In the present study, the maximum variation of ±2 mm was observed when overlay of CT reconstruction was carried out with the orthogonal reconstruction with respect to point A and all the active dwell positions. The shift may be attributed to the movement of the applicator while shifting the patient from the simulator room to the CT room for imaging. In our center, simulator and CT rooms are adjacent to each other, and the external reproducibility of the applicator with respect to the patient's leg position was maintained so that the movement of the applicator could be minimized. Sauer *et al.*[21] concluded that geometrical uncertainties such as mobility of the rectum, was estimated to be less than 3 mm before and after treatment. The uncertainty increases with increasing time between the application and the treatment. Thomadsen *et al.*[22] concluded that no movement of the patient should be allowed because even small changes in the position of the legs can produce large change in the dose to the bladder and rectum. Grigsby *et al.*[23] reported movement of ICRU bladder and rectal reference points and Manchester points A and B relative to bony structures during a time interval of two intracavitary implants. It was concluded that the mean shift of about 10-15 mm was observed with dose deviation as large as 35%.

The successful implementation of image-based dosimetry for intracavitary brachytherapy for carcinoma of cervix depends on the accurate delineation of the critical structures and the target volume. The use of metal applicators in the present study produced artifacts that made delineation of the critical structures difficult to some extent. However, the use of radiopaque gauze pack in the vagina helped to delineate rectum, and contrast medium in the bladder enabled to differentiate the bladder from the cervix and the vagina. Other imaging modalities such as Magnetic Resonance imaging/Positron Emission Tomography (MRI/PET)-based volume delineation would improve the accuracy of delineation of critical structures and the target volume, as reported.[24–26]

Significant advances in imaging and planning systems have resulted in better evaluation and understanding of brachytherapy in carcinoma cervix. However, image-based brachytherapy is still not widely used in routine clinical practice due to various limitations. Metal applicators produce streak artifacts in the CT images - which makes reconstruction of the applicator difficult, which may lead to inaccurate applicator reconstruction. MRI/CT-compatible applicators are expensive and not as strong as metal applicators, which prohibits the use of these expensive applicators in routine clinical practice, especially in developing countries. Hence refinement of the existing applicators and development of new cost-effective applicators and fast reconstruction methods with delineation of targets and critical structures are required for the implementation of image-based brachytherapy in routine clinical practice.

## Conclusion

Our dosimteric study suggests that comparison of orthogonal X-ray-based and CT-based HDR ICA planning is feasible. ICRU rectal point dose correlates well with maximum rectal dose, while ICRU bladder point underestimates the maximum bladder dose. Further, incorporation of newer imaging modalities, refinements in applicators and planning systems and wider acceptability of conformal brachytherapy may revolutionize brachytherapy practice in carcinoma cervix.
